# Dual suppression of inner and outer mitochondrial membrane functions augments apoptotic responses to oncogenic MAPK inhibition

**DOI:** 10.1038/s41419-017-0044-1

**Published:** 2018-01-18

**Authors:** Madhavika N. Serasinghe, Jesse D. Gelles, Kent Li, Lauren Zhao, Franco Abbate, Marie Syku, Jarvier N. Mohammed, Brateil Badal, Cuahutlehuanitzin A. Rangel, Kyle L. Hoehn, Julide Tok Celebi, Jerry Edward Chipuk

**Affiliations:** 1 Department of Oncological Sciences, Icahn School of Medicine at Mount Sinai, One Gustave L. Levy Place, Box 1130, New York, NY 10029 USA; 2 The Tisch Cancer Institute, Icahn School of Medicine at Mount Sinai, One Gustave L. Levy Place, Box 1130, New York, NY 10029 USA; 3 Department of Dermatology, Icahn School of Medicine at Mount Sinai, One Gustave L. Levy Place, Box 1130, New York, NY 10029 USA; 4 The Graduate School of Biomedical Sciences, Icahn School of Medicine at Mount Sinai, One Gustave L. Levy Place, Box 1130, New York, NY 10029 USA; 5 Department of Pathology, Icahn School of Medicine at Mount Sinai, One Gustave L. Levy Place, Box 1130, New York, NY 10029 USA; 6 School of Biotechnology and Biomolecular Sciences, University of New South Wales, Sydney, NSW 2052 Australia; 7 The Diabetes, Obesity, and Metabolism Institute, Icahn School of Medicine at Mount Sinai, One Gustave L. Levy Place, Box 1130, New York, NY 10029 USA

## Abstract

Mitogen-activated protein kinase (MAPK) pathway inhibitors show promise in treating melanoma, but are unsuccessful in achieving long-term remission. Concordant with clinical data, BRAF^V600E^ melanoma cells eliminate glycolysis upon inhibition of BRAF^V600E^ or MEK with the targeted therapies Vemurafenib or Trametinib, respectively. Consequently, exposure to these therapies reprograms cellular metabolism to increase mitochondrial respiration and restrain cell death commitment. As the inner mitochondrial membrane (IMM) is sub-organellar site of oxidative phosphorylation (OXPHOS), and the outer mitochondrial membrane (OMM) is the major site of anti-apoptotic BCL-2 protein function, we hypothesized that suppressing these critical mitochondrial membrane functions would be a rational approach to maximize the pro-apoptotic effect of MAPK inhibition. Here, we demonstrate that disruption of OXPHOS with the mitochondria-specific protonophore BAM15 promotes the mitochondrial pathway of apoptosis only when oncogenic MAPK signaling is inhibited. Based on RNA-sequencing analyses of nevi and primary melanoma samples, increased pro-apoptotic BCL-2 family expression positively correlates with high-risk disease suggesting a highly active anti-apoptotic BCL-2 protein repertoire likely contributes to worse outcome. Indeed, combined inhibition of the anti-apoptotic BCL-2 repertoire with BH3-mimetics, OXPHOS, and oncogenic MAPK signaling induces fulminant apoptosis and eliminates clonogenic survival. Altogether, these data suggest that dual suppression of IMM and OMM functions may unleash the normally inadequate pro-apoptotic effects of oncogenic MAPK inhibition to eradicate cancer cells, thus preventing the development of resistant disease, and ultimately, supporting long-term remission.

## Introduction

Mutations within the mitogen-activated protein kinase (MAPK) pathway are bona fide oncogenes that are responsible for ~ 80% of melanoma cases^1,2^. This pathway is mutated at distinct steps (e.g., RAS^G12V^ and BRAF^V600E^) leading to constitutive hyper-activation of pro-survival/pro-proliferation signaling in the absence of extracellular ligands^3–7^. As such, significant efforts have focused on the identification and investigation of targeted therapies to inhibit oncogenic MAPK signaling, including PLX4032 (Vemurafenib; BRAF^V600E^ inhibitor) and GSK1120212 (Trametinib; MEK inhibitor)^8–11^. While short-term disease stabilization occurs in a majority of patients, drug-resistant disease rapidly ensues through a variety of molecular mechanisms^11–14^. These clinical observations suggest that novel therapeutic strategies to enhance cancer cell killing are required to increase primary clinical responses to targeted therapies, and thwart selection and/or expansion of therapy-resistant populations.

Oncogenic MAPK signaling in melanoma leads to metabolic reprogramming and dependency upon glycolysis and glutaminolysis for rapid proliferation^15^. Likewise, recent reports suggest that inhibition of oncogenic MAPK signaling increases mitochondrial biogenesis and oxidative phosphorylation (OXPHOS) to sustain ATP generation^16^. BRAF^V600E^ melanoma in situ and various melanoma models respond to PLX4032 or GSK1120212 treatment by eliminating glycolysis and become dependent upon mitochondrial respiration to maintain survival^15^. Through distinct, yet complementary mechanisms, BRAF^V600E^ or MEK inhibition leads to PGC1α-dependent expansion of the mitochondrial network and increased mitochondrial fusion to enhance the efficiency of OXPHOS^16,17^. Likewise, in a variety of human cancer cell lines and primary melanoma tissues, oncogenic MAPK signaling promotes chronic mitochondrial division by ERK1/2-dependent phosphorylation and activation of the mitochondrial division GTPase, dynamin related protein 1 (DRP1), at serine 616^17,18^. DRP1 serine 616 phosphorylation is sufficient to actuate mitochondrial dysfunction in transformed cells, dichotomize wild-type BRAF (BRAF^Wt^) and BRAF^V600E^ melanoma tissues, and also broadly impacts upon cancers arising from the brain, breast, and pancreas^17–20^. These observations suggest that oncogenic MAPK signaling regulates mitochondrial biology and OXPHOS during tumorigenesis and after therapeutic interventions.

While the inner mitochondrial membrane (IMM) coordinates the assembly and function of the electron transport chain and OXPHOS complexes, the outer mitochondrial membrane (OMM) controls apoptosis by governing the B-cell lymphoma 2 (BCL-2) family of proteins^21,22^. Apoptosis proceeds when the BCL-2 family compromises the OMM allowing for cytochrome *c* to gain access to the cytoplasm, which leads to caspase activation and the subsequent hallmark features of apoptosis^21,23^. Anti-apoptotic BCL-2 proteins (e.g., BCL-2, BCL-xL, MCL-1) preserve survival by directly inhibiting the pro-apoptotic BCL-2 proteins^23^. The pro-apoptotic proteins are divided into ‘‘effectors’’ and the ‘‘BH3-only’’ members. The effector proteins BAK and BAX homo-oligomerize into proteolipid pores at the OMM to release cytochrome *c* through a process referred to as mitochondrial outer membrane permeabilization (MOMP)^21^. In order for BAK/BAX to promote MOMP, they require transient interactions with the BH3-only proteins, BID, and BIM^24–26^. The biochemical decision to induce MOMP depends on the availability of BID and BIM to activate BAK/BAX, which is dictated by the availability of the anti-apoptotic BCL-2 reserve to sequester all pro-apoptotic BCL-2 proteins^23^. In the clinic, small molecules that inhibit the anti-apoptotic BCL-2 proteins quench this reserve and lower the cellular threshold to initiate MOMP. This class of compounds is referred to as ‘‘BH3 mimetics’’ and examples include: ABT199 (Venetoclax, BCL-2 inhibitor), ABT263/ABT737 (Navitoclax, BCL-2/BCL-xL inhibitor), and A1210477 (pre-clinical compound, MCL-1 inhibitor), some of which have gained breakthrough status for hematological malignancies^27–31^.

Despite suggestions in the literature that targeting OXPHOS may be a rational means to enhance the anti-cancer effects of PLX4032 and GSK1120212, clinically relevant molecules with mechanistic integration into broader mitochondrial functions (e.g., MOMP and apoptosis) are not defined. Here, we investigated how the dual suppression of two critical mitochondrial membrane functions: OXPHOS and the anti-apoptotic BCL-2 repertoire at the IMM and OMM, respectively, impact upon the pro-apoptotic effects of oncogenic MAPK inhibition.

## Results

**Fig. 1:**
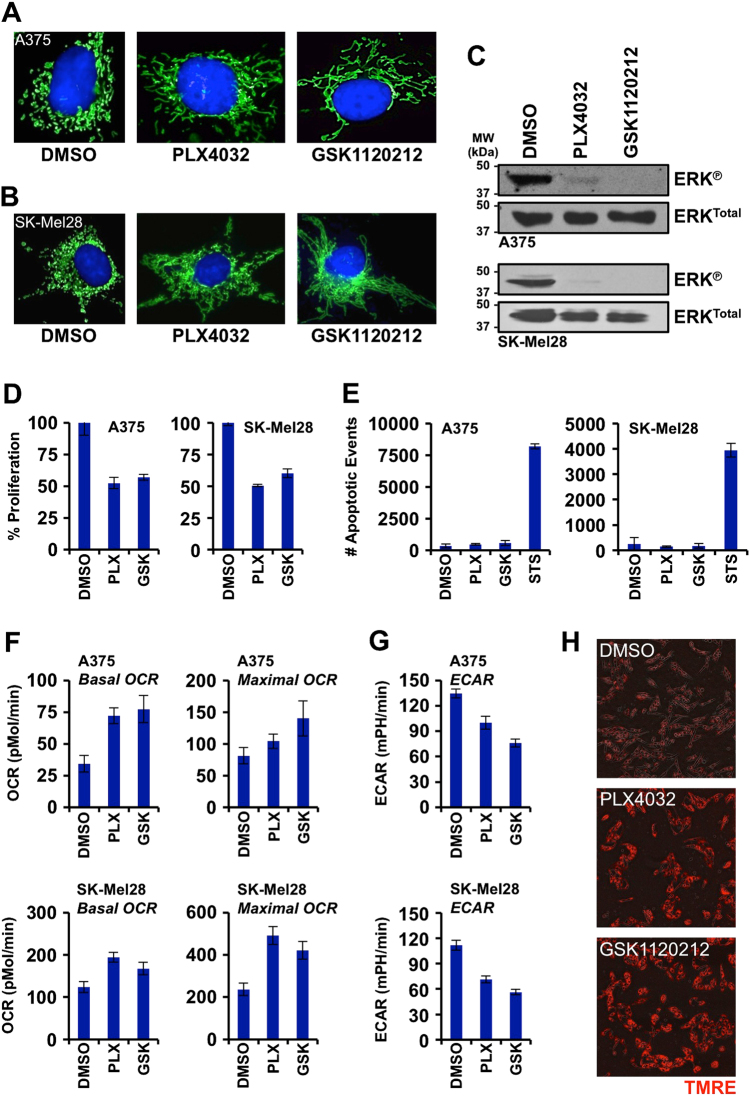
Inhibition of oncogenic MAPK signaling promotes mitochondrial respiration **a**,** b** A375 and SK-Mel28 were treated with PLX4032 (1 μM) or GSK1120212 (10 nM) for 24 h, loaded with MitoTracker Green and Hoechst 33342, and live-cell imaged. **c** A375 and SK-Mel28 were treated as in *A*, and cell lysates were subjected to SDS-PAGE and western blot for indicated proteins. **d** A375 and SK-Mel28 were treated as in *A* for 24 h, and proliferation was quantified by IncuCyte ZOOM. Endpoint data for 24 h are shown. **e** A375 and SK-Mel28 were treated as in *A* for 24 h, and the number of apoptosis events was captured by IncuCyte ZOOM. Endpoint data for 24 h are shown. Staurosporine (STS, 100 nM) is a potent inducer of apoptosis, and a positive control. **f** Basal and maximal mitochondrial oxygen consumption rates (OCR) were determined by Seahorse XF96 analyses. Cells were treated with PLX4032 (1 μM) or GSK1120212 (10 nM) for 24 h before analyses. OCR values were normalized for cell number. **g** Extracellular acidification rates (ECAR) were determined by Seahorse XF96 analyses. Cells were treated with PLX4032 (1 μM) or GSK1120212 (10 nM) for 24 h before analyses. ECAR values were normalized for cell number. **h** TMRE-loaded A375 were treated as in *A* for 24 h, and Δ*φ*_M_ was captured by IncuCyte ZOOM. Endpoint images containing bright field and TMRE channels at 24 h are shown. All data are representative of at least triplicate experiments, and reported as ± S.D., as required

Our investigations began by examining if the chosen cellular model systems responded to PLX4032 and GSK1120212 treatments. For the majority of this study, we evaluated two human BRAF^V600E^ melanoma cell lines derived from metastatic disease: A375 and SK-Mel28. These melanoma cell lines were recently shown to have highly fragmented mitochondrial networks, which can be corrected by the inhibition of oncogenic MAPK signaling leading to mitochondrial fusion^17^. To observe this phenotype, we treated A375 and SK-Mel28 with PLX4032 or GSK1120212, loaded these cells with MitoTracker Green and Hoechst 33342 (i.e., mitochondrial and nuclear labels, respectively), and performed live-cell imaging. In both cell lines treated with either drug, rapid mitochondrial fusion was observed in the vast majority of cells (Fig. 1a, b; A375 = 95% of cells with fused mitochondrial networks, SK-Mel28 = 72% of cells with fused mitochondrial networks; below 15% for all DMSO-treated cells). To confirm that oncogenic MAPK signaling was inhibited following the above treatments, we performed sodium dodecyl sulfate polyacrylamide gel electrophoresis (SDS-PAGE) and western blot analyses for activated ERK (p44/42), and indeed PLX4032 and GSK1120212 inhibited ERK phosphorylation in A375 and SK-Mel28 (Fig. 1c). Likewise, these drug treatments rapidly reduced proliferation (Fig. 1d and Supplementary Fig. S1C), and no increase in apoptosis was detected (Fig. 1e and Supplementary Fig. S1A, B). Altogether, these data indicate the cell lines, treatments, and responses are appropriate to further this study.

A suggested response to PLX4032- or GSK1120212-induced mitochondrial fusion is increased mitochondrial respiration. To examine this phenotype, we performed Seahorse analyses to determine the basal and maximal respiratory rates following PLX4032 or GSK1120212 treatments. While the cell lines demonstrated specific patterns of increased oxygen consumption rates (OCR): A375 cells doubled their basal OCR; SK-Mel28 cells doubled their maximal OCR; overall, both A375 and Sk-Mel28 demonstrated increased basal and maximal OCR following the inhibition of oncogenic MAPK signaling (Fig. 1f). In parallel to increased OCR, both cell lines treated with either drug decreased their extracellular acidification rates (ECAR), indicative of reduced glycolysis (Fig. 1g), as expected from both basic science and clinical investigations with targeted therapies against MAPK signaling. Finally, we also confirmed the increased OCR with a mitochondrial potentiometric fluorescent dye, tetramethylrhodamine ethyl ester (TMRE), in living cells using fluorescent microscopy. A375 treated with either PLX4032 or GSK1120212 displayed enhanced TMRE fluorescence, indicative of increased mitochondrial function (Fig. 1h and Supplementary Fig. S1D). Similar data with SK-Mel28 were also obtained (Fig. S1D, E).

We hypothesized that the increase in mitochondrial respiration maintains cellular survival when targeted therapies decrease glycolysis. To test our hypothesis, we were interested in a non-toxic small molecule that inhibits mitochondrial OXPHOS to combine with targeted therapies, and then examine cellular survival. From the literature, we identified a second-generation mitochondrial uncoupling agent, BAM15, which specifically inhibits OXPHOS^32^. BAM15 has minimized off-target effects like standard mitochondrial uncoupling agents (e.g., carbonyl cyanide-4-phenylhydrazone, FCCP, one side effect is plasma membrane disruption), and is well tolerated in vivo^32^.

**Fig. 2:**
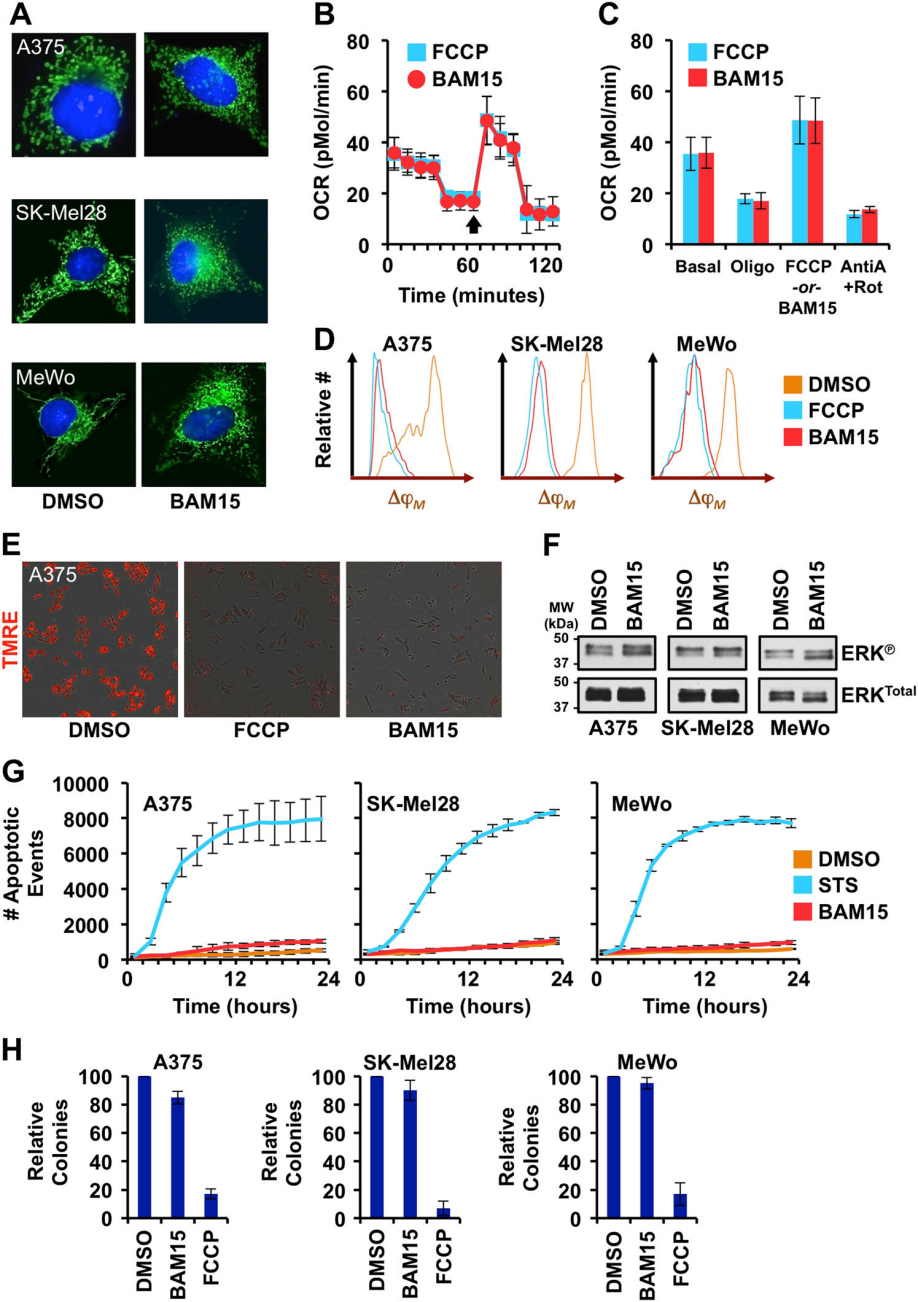
BAM15 uncouples OXPHOS from electron transport in melanoma cell lines **a** A375, SK-Mel28, and MeWo were treated with DMSO or BAM15 (10 μM) for 24 h, loaded with MitoTracker Green and Hoechst 33342, and live-cell imaged. **b**–**c** A375 were analyzed by Seahorse mitochondrial stress test. FCCP (1 μM) or BAM15 (1 μM) were added (black arrow) to compare uncoupling efficiency. **d** A375 cells were treated with FCCP (10 μM) or BAM15 (10 μM) for 2 h, loaded with TMRE (100 nM), and analyzed by flow cytometry. **e** Same as **d**, but analyzed by IncuCyte ZOOM. **f** A375, SK-Mel28, and MeWo were treated with BAM15 (10 μM) for 24 h, and cell lysates were subjected to SDS-PAGE and western blot for indicated proteins. **g** A375, SK-Mel28, and MeWo were treated with BAM15 (10 μM) or staurosporine (1 μM, positive control) for 24 h, and the kinetics of cell death were detected using IncuCyte ZOOM. **h** A375, SK-Mel28, and MeWo were treated with BAM15 (10 μM) or FCCP (10 μM) for 24 h; colony formation was quantified 7 days later. All data are representative of at least triplicate experiments, and reported as ± S.D., as required

To begin, we analyzed the mitochondrial and cellular effects of BAM15 treatment as a single agent. A375 and SK-Mel28 were treated with BAM15, and then the cells were loaded with MitoTracker Green and Hoechst before live-cell imaging. BAM15 treatment led to marked mitochondrial fragmentation beyond the chronic levels observed in BRAF^V600E^ melanoma lines (Fig. 2a); and in a metastatic melanoma line with BRAF^Wt^ and a normal mitochondrial network (MeWo), mitochondrial fragmentation was also observed (Fig. 2a). BAM15 is described to function similar to FCCP, and we determined if this is correct using several assays^32^. First, we examined if BAM15 treatment uncoupled mitochondrial membrane potential to engage maximal respiration in melanoma cell lines. Using the Seahorse mitochondrial stress test, the rates of spare mitochondrial capacity of A375 cells were analyzed following FCCP or BAM15 treatments. For all OCR measurements, before (i.e., basal respiration and oligomycin-stimulated) and after BAM15 or FCCP treatments (i.e., uncoupled, and non-mitochondrial after Antimycin A + Rotenone-inhibited), OCRs were nearly identical suggesting that BAM15 was equally efficient at mitochondrial uncoupling compared to FCCP (Fig. 2b, c). Next, we compared changes to mitochondrial inner membrane potential (Δψ_M_) following exposure to BAM15 and FCCP using cells loaded with TMRE and flow cytometry and live-cell imaging; both assays revealed nearly similar rates and extent of changes to Δψ_M_ suggesting BAM15 is functionally equivalent to FCCP (Fig. 2d, e and Supplementary Fig. S2A). Finally, the durability of each drug was compared by analyzing mitochondrial uncoupling (maximal OCR), and BAM15 maintains >80% activity compared to FCCP (Supplementary Fig. S2D). Data presented in Fig. 2b–e use A375, but SK-Mel28 exhibited identical responses (Supplementary Fig. S2B, C).

To ensure no off-target effects in our cellular models, we also examined if BAM15 affected oncogenic MAPK signaling to avoid subsequent confounding interpretations. A375, SK-Mel28, and MeWo were treated with BAM15, whole-cell lysates were analyzed for ERK phosphorylation by SDS-PAGE and western blot, and no changes in ERK phosphorylation were detected (Fig. 2f). In vivo experimentation using mice suggests BAM15 should have minimal cellular toxicity, and we determined if BAM15 regulated survival and colony formation with all the cell lines. Indeed, BAM15 treatment for 24 h led to no detectible cell death (Fig. 2g; staurosporine treatment is a positive control for cell death), or significant changes in colony formation (Fig. 2h; FCCP treatment caused a marked decrease in colony formation suggesting toxicity). Altogether, these data support that BAM15 is a highly efficient mitochondrial uncoupling agent, with minimal off-target effects and influences on cellular survival.

**Fig. 3:**
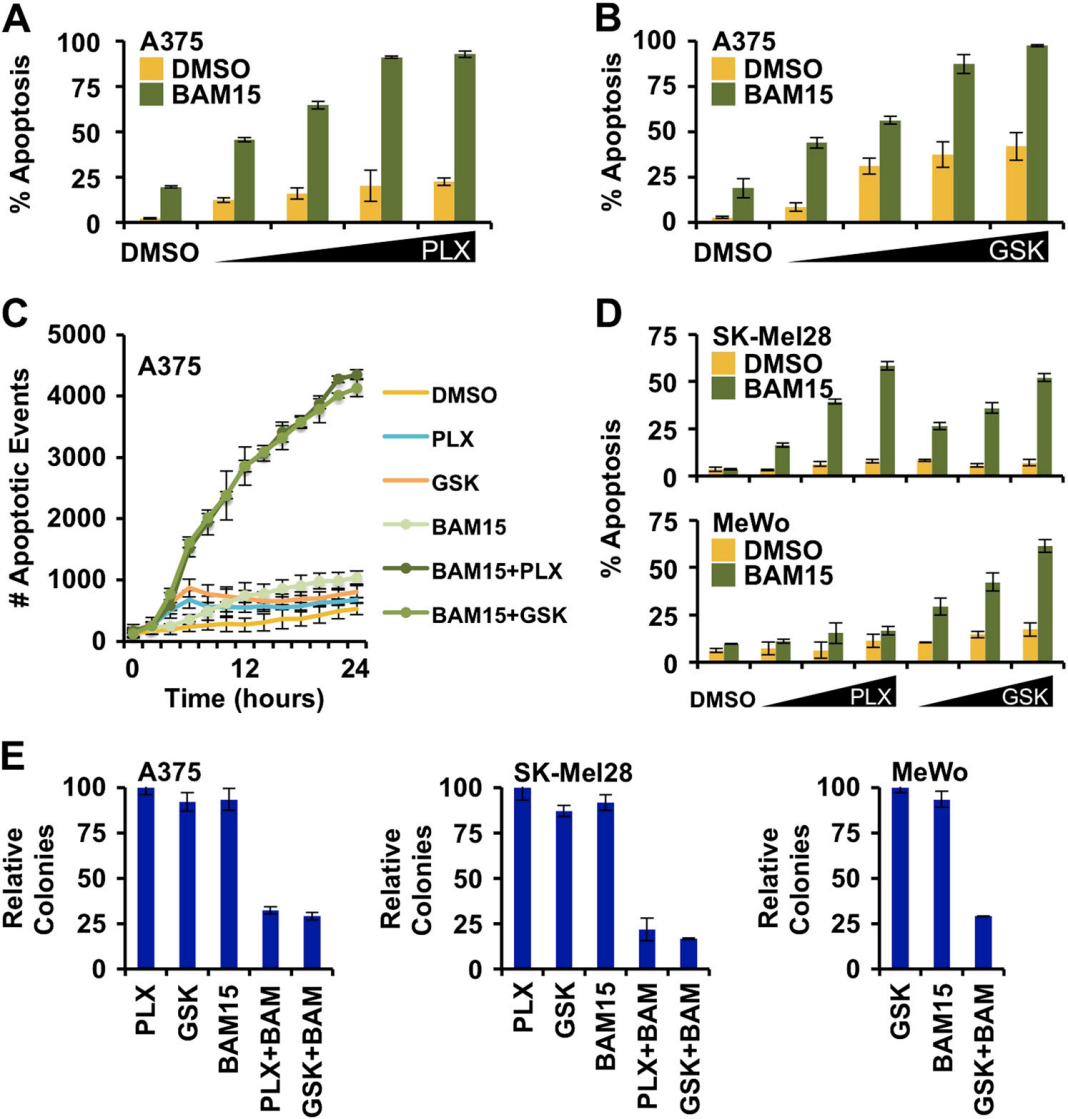
BAM15 promotes cell death when oncogenic MAPK signaling is inhibited **a** A375 were treated with increasing doses of PLX4032 (0.5, 1, 10, 20 μM) ± BAM15 (10 μM) for 24 h, and apoptosis was detected by AnnexinV-FITC staining and flow cytometry. **b** Same as **a**, but GSK1120212 (1, 5, 10, 25 nM). **c** A375 were treated with combinations of PLX4032 (0.5 μM), GSK1120212 (1 nM), and BAM15 (10 μM); the kinetics of cell death were detected using an IncuCyte ZOOM. **d** Same as **a** and **b**, but with SK-MEL-28 (top panel) and MeWo (bottom panel). **e** A375, SK-Mel28, and MeWo were treated with combinations of PLX4032 (0.5 μM), GSK1120212 (1 nM), and BAM15 (10 μM) for 24 h; colony formation was quantified 7 days later. All data are representative of at least triplicate experiments, and reported as ± S.D., as required

Next, we determined if BAM15-induced inhibition of OXPHOS promoted cell death when oncogenic MAPK signaling is ablated with PLX4032 or GSK1120212. Based on results presented in Fig. 2, we treated A375 cells with BAM15 (10 μM, or DMSO) in the presence of increasing doses of PLX4032 or GSK1120212, and assessed cell death responses 24 h later by flow cytometry. As single agents, PLX4032 and GSK1120212 failed to induce marked cell death, yet in the presence of BAM15, both drugs induced significant apoptosis that was both dose-dependent and time-dependent (Fig. 3a–c). The same results were obtained for SK-Mel28 (Fig. 3d, top, S3A); and MeWo responded to GSK1120212 + BAM15, and not PLX4032 + BAM15, suggesting the resulting cell death was due to on-target effects (Fig. 3d, bottom, S3B). Indeed, all treatments that led to marked apoptosis also resulted in a ~ 75% reduction in colony formation (Fig. 3e). These data suggest that the inhibition oncogenic MAPK signaling enhances cell death responses when OXPHOS is blocked by BAM15.

**Fig. 4:**
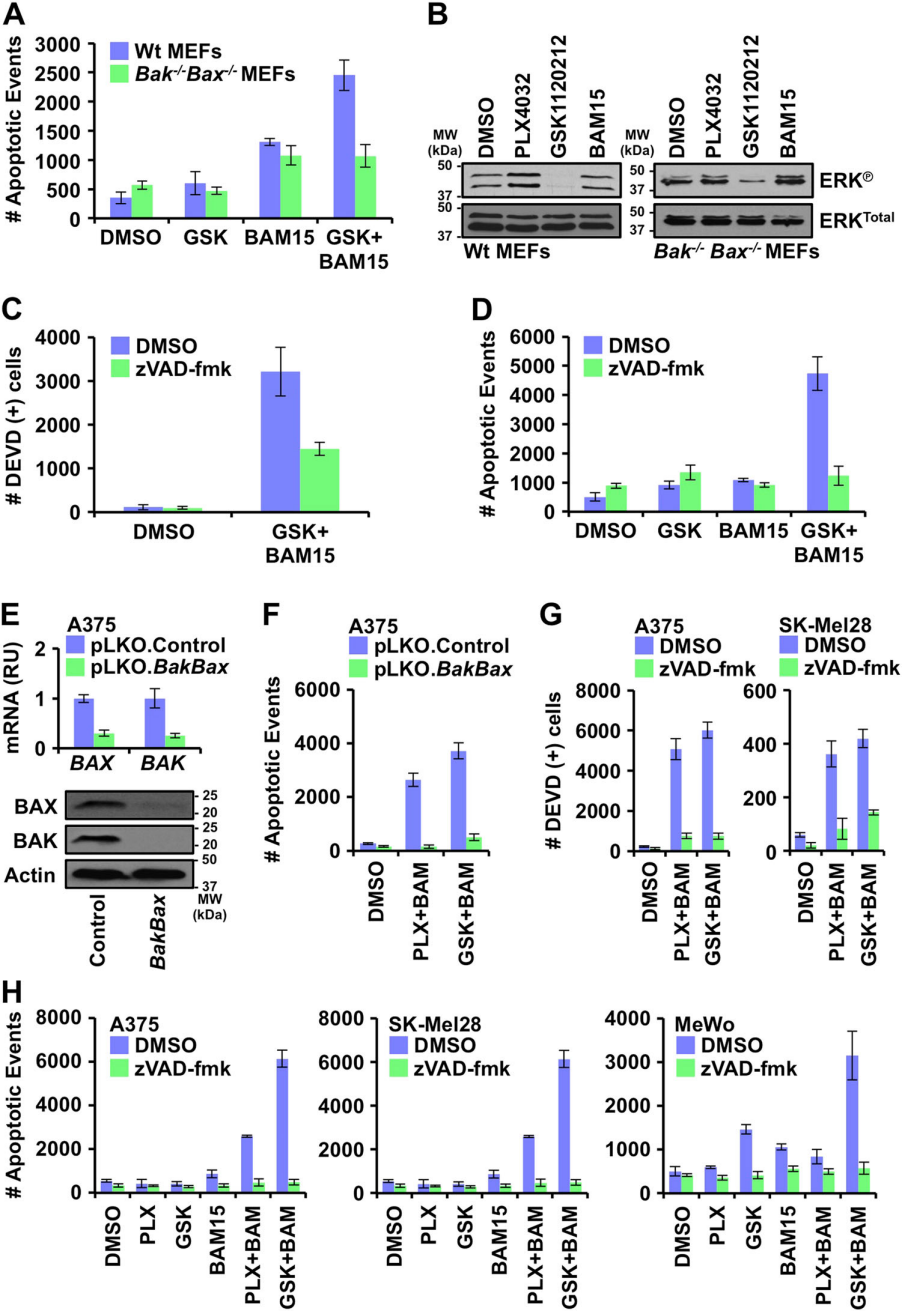
Combined inhibition of OXPHOS and oncogenic MAPK signaling induces the mitochondrial pathway of apoptosis **a** Wt and *Bak*^*-/-*^*Bax*^*-/-*^ MEFs were treated with GSK1120212 (10 nM) ± BAM15 (10 μM) for 36 h, and apoptosis was detected by AnnexinV-FITC staining with an IncuCyte Zoom. Endpoint data for 24 h are shown. **b** Wt and *Bak*^*-/-*^*Bax*^*-/-*^ MEFs were treated with PLX4032 (1 μM), GSK1120212 (10 nM), or BAM15 (10 μM) for 24 h, and cell lysates were subjected to SDS-PAGE and western blot for indicated proteins. **c** Wt MEFs were treated with GSK1120212 (10 nM), BAM15 (10 μM), and ± zVAD-fmk (100 μM) for 36 h, and caspase activity was detected using a DEVD-FITC peptide reporter with an IncuCyte ZOOM. Endpoint data for 36 h are shown. **d** Wt MEFs were treated with GSK1120212 (10 nM), BAM15 (10 μM), and ± zVAD-fmk (100 μM) for 36 h, and apoptosis was detected by AnnexinV-FITC staining with an IncuCyte ZOOM. Endpoint data for 36 h are shown. **e** A375 expressing shRNAs against *BAK* and *BAX* were harvested for qPCR (relative units, RU) and SDS-PAGE/western blot analyses of indicated mRNAs and proteins, respectively. **f** A375 expressing shRNAs against *BAK* and *BAX* were treated with PLX4032 (1 μM) or GSK1120212 (10 nM), ± BAM15 (10 μM); apoptosis was detected by AnnexinV-FITC staining with an IncuCyte ZOOM. Endpoint data for 36 h are shown. **g** A375 and SK-Mel28 were treated with indicated combinations of PLX4032 (1 μM) or GSK1120212 (10 nM), BAM15 (10 μM), ± zVAD-fmk (100 μM), and caspase activity was detected by DEVD-FITC with an IncuCyte ZOOM. Endpoint data for 36 h are shown. **h** A375, SK-Mel28, and MeWo were treated with indicated combinations of PLX4032 (1 μM), GSK1120212 (10 nM), and/or BAM15 (10 μM) for 24 h, and apoptosis was detected by AnnexinV-FITC staining with an IncuCyte ZOOM. Endpoint data for 24 h are shown. All data are representative of at least triplicate experiments, and reported as ± S.D., as required

To better understand the mechanism of cell death in Fig. 3, we compared genetically matched SV40-transformed wild type (Wt) mouse embryonic fibroblasts (Wt MEFs) and MEFs deficient for *Bak* and *Bax* (*Bak*^*-/-*^*Bax*^*−/−*^ MEFs). *Bak*^*-/-*^*Bax*^*-/-*^ MEFs are refractory to inducers of apoptosis, and, therefore, are useful to define if signaling events and drugs induce the mitochondrial pathway of apoptosis^33^. Wt MEFs do not have BRAF^V600E^, but require active MEK signaling for homeostasis, so we examined GSK1120212 ± BAM15. Wt and *Bak*^*-/-*^*Bax*^*-/-*^ MEFs were treated with GSK1120212 (10 nM) ± BAM15 (10 μM) for 36 h, and apoptosis was measured using a recently published real-time Annexin V-based high-content live-cell imaging technique. Single treatments of GSK1120212 or BAM15 resulted in minimal cell death, but in combination, marked apoptosis was observed in only Wt MEFs suggesting BAK and BAX are required for death (Fig. 4a and Supplementary Fig. S4A, B). Phosphorylated ERK was also examined after drug treatment to ensure GSK1120212 inhibited ERK signaling in both MEF lines; and GSK1120212 turned off ERK signaling, whereas BAM15 or PLX4032 exposure did not inhibit the ERK pathway (Fig. 4b).

Moreover, the mitochondrial pathways of apoptosis is usually delayed and/or blocked when the enzymatic activity of caspases is inhibited, and, therefore, we analyzed if GSK1120212 ± BAM15-induced cell death required caspase activity^34,35^. Wt MEFs were pre-treated with zVAD-fmk (100 μM), a cell permeable irreversible pan-caspase inhibitor, prior to GSK1120212 ± BAM15 treatment for 24 h. The presence of zVAD-fmk prevented the majority of caspase activity (measured through DEVD cleavage) and apoptosis induced by GSK1120212 ± BAM15 (Fig. 4c, d and Supplementary Fig. S4C). Using similar approaches, and extending to PLX4032  ± BAM15, the melanoma cell lines showed similar phenotypes and requirements for BAK/BAX and caspases to undergo cell death (Fig. 4e–h and Supplementary Fig. S4D, E). For example, we silenced the expression of both *BAK* and *BAX* in A375 by shRNAs to interrogate the requirement of the mitochondrial pathway of apoptosis (Fig. 4e; other cell lines are data not shown). Altogether, these observations suggest that BAM15-induced inhibition of OXPHOS promotes the mitochondrial pathway of apoptosis when oncogenic MAPK signaling is ablated with PLX4032 or GSK1120212.

**Fig. 5:**
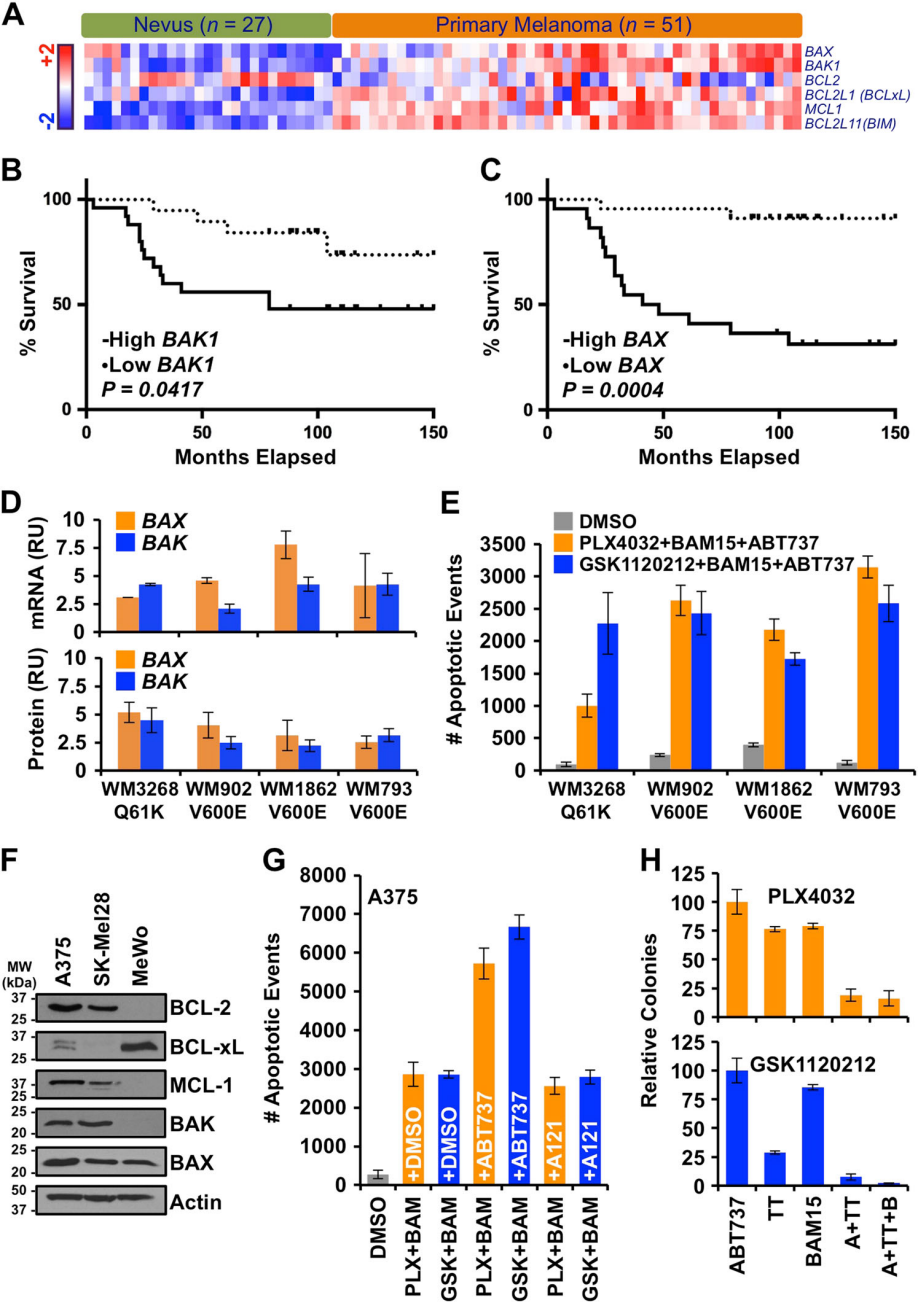
Dual suppression of inner and outer mitochondrial membrane functions augments pro-apoptotic responses to oncogenic MAPK signaling inhibition **a** Pro-apoptotic and anti-apoptotic genes differentially expressed between human nevus samples (*n* = 27) and primary melanomas (*n* = 51). Differential expression is defined by at least a 1.5 linear fold change and a *q*-value less than 0.005. Protein-coding mRNA with counts greater than 10 per million in at least two samples were considered. Heatmap represents *z*-scores of expression from RNA-seq data^36^. Hierarchical clustering was performed for both samples, *n* = 78 and genes, *n* = 6. **b–c** Kaplan–Meyer curves representing the 5-year overall survival for *BAK1* (*P* = 0.0417) and *BAX* (*P* = 0.0004) across primary melanoma samples (*n* = 51). ‘‘High’’ (*n* = 24) and ‘‘low’’ samples (*n* = 27) were defined based on high (positive) and low (negative) *z*-scores generated from gene expression data. Log-rank test was utilized to determine statistical significance. **d** WM3268, WM902B, WM1862, and WM793 were harvested for qPCR (relative units, RU) (upper panel); and SDS-PAGE/western blot analyses of indicated mRNAs and proteins (lower panel), respectively. All mRNA and protein expression data were normalized to melanocytes (RU = 1). **e** WM3268, WM902B, WM1862, and WM793 were treated with PLX4032 (1 μM) or GSK1120212 (10 nM), in the presence of BAM15 (10 μM) and ABT737 (1 μM) for 24 h, and apoptosis was detected by AnnexinV-FITC staining with an IncuCyte ZOOM. Endpoint data for 36 h are shown. **f** A375, SK-Mel28, and MeWo whole-cell lysates were subjected to SDS-PAGE and western blot for indicated proteins. Actin is the loading control. **g** A375 were treated with PLX4032 (1 μM) or GSK1120212 (10 nM), in the presence of BAM15 (10 μM), ± ABT737 (1 μM) or ± A1210477 (1 μM); apoptosis was detected by AnnexinV-FITC staining with an IncuCyte ZOOM. Endpoint data for 36 h are shown. **h** A375 were treated with indicated combinations of ABT737 (1 μM), targeted therapy (TT: 1 μM PLX4032 or 10 nM GSK1120212), and BAM15 (10 μM) for two weeks, with drugs replaced every 48 h. Cells were then cultured for an addition two weeks, and colony formation was quantified. All data are representative of at least triplicate experiments, and reported as ± S.D., as required

As the BAK/BAX-dependent mitochondrial pathway of apoptosis is responsible for BAM15-mediated cell death when oncogenic signaling is inhibited, we determined the expression of *BAK* and *BAX* in human melanoma and their relationship to overall survival. We compared the expression of pro-apoptotic (i.e.,* BAK, BAX, BIM*) and anti-apoptotic genes (i.e.,* BCL-2, BCL-xL, MCL-1*) differentially expressed between human nevus samples (*n* = 27) and primary melanomas (*n* = 51)^36^. Differential expression was defined by at least a 1.5 linear fold change and a *q*-value less than 0.005, and these data were derived from a recently published study from our group (Fig. 5a)^36^. Interestingly, increased expression of *BAK* and *BAX* strongly correlated with decreased overall survival (Fig. 5b, c), whereas the remaining genes demonstrated less impressive correlations (Supplementary Fig. S5A). These observations suggested that primary melanoma lesions are functionally competent to induce BAK/BAX-dependent apoptosis, but perhaps actively inhibit MOMP and cell death responses due to sufficiently tonic anti-apoptotic function.

To investigate this hypothesis, we evaluated four primary human melanoma cells lines: WM3268 (RAS^Q61K^), WM902B (BRAF^V600E^), WM1862 (BRAF^V600E^), and WM793 (BRAF^V600E^). First, we validated that BAK and BAX mRNA and protein expressions were elevated compared to pre-malignant primary melanocytes by quantitative PCR (qPCR) and western blot, respectively (Fig. 5d). Next, we determined which anti-apoptotic BCL-2 family proteins are expressed in these cell lines by western blot, and noted that the majority had significant levels of BCL-2 and BCL-xL, along with MCL-1 (Supplementary Fig. S5B). Therefore, we predicted that ABT737, a small molecule BH3 mimetic that inhibits BCL-2 and BCL-xL would be an appropriate tool to provoke cell death responses in these cell lines. While ABT737 had minimal effects as a single treatment, as did PLX4032, GSK1120212, and BAM15 (Supplementary Fig. S5C, D); BAM15 promoted apoptosis in the presence of PLX4032 (or GSK1120212), and this response was markedly enhanced by the addition of ABT737—suggesting anti-apoptotic BCL-2 and BCL-xL actively regulate these apoptotic responses (Fig. 5e and Supplementary Fig. S5C). Furthermore, we similarly evaluated the metastatic lines for BCL-2 family expression, and each had a unique pattern of anti-apoptotic BCL-2 proteins (Fig. 5f). For A375, we compared ABT737 and A1210477 (BH3 mimetic specific to MCL-1) for the enhancement of PLX4032/GSK1120212 + BAM15-induced apoptosis. As shown, ABT737 increased apoptotic responses by 100%, while A1210477 failed to enhance cell killing suggesting that MCL-1 inhibition is not crucial to successful treatment of naive A375 cells (Fig. 5g).

Finally, resistance to targeted therapies like PLX4032 and GSK1120212 is the major source of treatment failure and melanoma relapse. Given the marked levels of apoptosis when PLX4032/GSK1120212 is combined with BAM15 and ABT737 (Fig. 5g and Supplementary Fig. S5C), we evaluated how this triple combination therapy impacted on drug resistance and colony outgrowth. A typical treatment course for PLX4032/GSK1120212 lasts several weeks to months dependent upon observable disease progression and/or toxicity. To model this situation, A375 cells were treated with individual drugs and combinations for two weeks (drugs replaced every 48 h), and then cultured in the absence of drugs for two more weeks, and the colony formation was quantified. Indeed, several of the individual drug treatments allowed for considerable colony outgrowth suggesting that ABT737, BAM15, and PLX4032 revealed minimal changes to clonogenic survival (Fig. 5h); in contrast, combinations of PLX4032/GSK11220212 with ABT737 and BAM15 eliminated the vast majority of colony outgrowth with the combination of GSK1120212 + ABT737 + BAM15 reducing drug-resistant outgrown by nearly 99% (Fig. 5h).

## Discussion

Recent literature indicates a crucial role for mitochondria function in tumor initiation and progression due to their central role in cellular energy production, biosynthesis of macromolecules, and apoptosis—all of which intersect with several cancer signaling pathways^37–39^. Furthermore, mitochondrial transcription and mitochondrial DNA contribute to metastasis and prognosis^40,41^. Here, we describe a novel therapeutic strategy, which targets multiple aspects of mitochondrial function alongside oncogenic MAPK pathway inhibition. Our data indicate that simultaneously targeting these distinct cellular processes results in a rapid apoptotic response and reduced opportunities for drug-resistant populations to arise.

In diverse models of cancer, the oncogenic RAS-MAPK pathway mediated cellular transformation depends on metabolic reprogramming, leading to high levels of glycolytic activity and compromised mitochondrial function (known as the Warburg effect)^15,42^. Likewise, blocking the oncogenic RAS-MAPK pathway using targeted inhibitors against BRAF^V600E^ or MEK reversed this metabolic phenotype to favor mitochondrial respiration^17,18^. In order to target the acquired dependency on mitochondrial function by MAPK inhibited melanoma cells, we utilized the novel mitochondrial membrane potential uncoupler BAM15 to induce proton leak and decrease ATP synthesis. BAM15 is a non-cytotoxic molecule that shows similar effect as FCCP without associated toxicity and off-target effects (Fig. 2b–h)^32^. While BAM15 by itself did not cause cell death or alter proliferation, the combined treatment with low concentrations of PLX4032 or GSK1120212 sensitized melanoma cells to rapid cell death (Fig. 3a–d).

The cell death observed initiates through the BCL-2 family-mediated mitochondrial pathway of apoptosis, another fundamental mitochondrial process that is altered in cancer (Fig. 4a–h). ABT737 is a small molecule BH3-mimetic designed to target the anti-apoptotic BCL-2 family proteins BCL-2 and BCL-xL^30^. Each of the primary and metastatic melanoma cell lines used in our study showed unique anti-apoptotic protein (e.g., BCL2, BCL-xL and/or MCL-1) expression signature that related with their response to BH3-mimetics (Supplementary Fig. S5A). This highlights the utility of profiling BCL-2 family proteins for selecting treatment modalities against naive and resistant melanomas^43^. While ABT737 alone was not sufficient to induce apoptosis in melanoma cells, which abundantly express BAX and BAK, combining ABT737 with MAPK inhibition and BAM15 resulted in massive apoptosis within a few hours (Supplementary Fig. S5A, B, D). Therefore, decreasing the apoptotic threshold using BH3-mimetics is a feasible strategy to further enhance cell death in MAPK pathway mutated melanoma cells when used in combination with a mitochondrial toxin. Surprisingly, the patient data presented in Fig. 5a–c indicate that the pro-apoptotic proteins, BAX and BAK have increased expression in primary melanomas compared to nevi, and this is correlated with poor prognosis. We also observed elevated levels of BAK and BAX expression in melanomas compared to primary melanocytes. These observations suggest that primary melanoma lesions are functionally competent to induce BAK/BAX-dependent apoptosis, but inhibit MOMP and cell death responses due to sufficiently tonic anti-apoptotic function^44^.

There are several advantages to the combination strategy described here^1^: The rapid induction of cell death eliminates the majority of cancer cells, while the remaining cells failed to proliferate; indicating that this combination allows minimal chances for drug-resistant cells to develop and/or persist (Fig. 5h)^2^; The specificity afforded by the targeted therapies (i.e., oncogenic MAPK inhibitors as well as BH3-mimetics) would minimize off-target toxicity; and^3^ Targeting three functional pathways required for cancer cell survival, proliferation, and drug response will likely reduce the generation of drug-resistant populations. Future studies using our proposed combinations should include both primary melanoma models and in vivo murine and patient-derived xenograft approaches.

Numerous studies have revealed that chronic mitochondrial division is associated with multiple features of oncogenic MAPK signaling, including cellular transformation, cancer like metabolism, tumorigenesis, cancer stem cells, and metastasis^17,18,20,45,46^. In these models, the mitochondrial fission protein DRP1 is activated by ERK1/2-mediated phosphorylation at serine 616 (DRP1^S616^) resulting in increased mitochondrial fission^17,19^. Our previous studies have also revealed that DRP1^S616^ phosphorylation has prognostic value for which BRAF^V600E^ positive skin lesions undergo malignant transformation^19^. We highlight the mechanistic contribution of DRP1 in melanoma because chronic mitochondrial division is a control point for responses to oncogenic MAPK signaling: including mitochondrial function, transformation, cellular metabolism, and apoptotic sensitivity. Understanding which parameters control oncogenic MAPK signaling associated phenotypes will lead to enhanced therapies that hone on individual disease rather than generalized treatments that do not show significant clinical success. Our work demonstrates that dual targeting of the inner and OMM function in combination with targeted MAPK pathway inhibition rapidly and effectively eliminates cancer cells. We predict that this treatment combination will drastically reduce drug-resistant disease and progression of malignant melanoma.

## Materials and methods

### Reagents

All cell culture reagents were from Invitrogen; and standard laboratory reagents were from Sigma-Aldrich or Fisher Scientific. Drugs were from: PLX4032, GSK1120212, A1210477 (Selleck); ABT737 (Abbvie); FCCP, Antimycin A, Staurosporine (Sigma-Aldrich), zVAD-fmk (VWR Scientific). Antibodies (clone or source): ERK^Total^ & ERK^℗^ (Cell Signaling); Actin (C4), BCL-2 (100), MCL-1 (Rockland), BAK (NT), BAX (N20), BCL-xL (S18). MitoTracker Green and TMRE were from Invitrogen, and Hoechst 33342 was from Anaspec.

### Cell culture, apoptosis assays, and clonogenic survival

A375, SK-MEL-28, MeWo, and SV40-transformed mouse embryonic fibroblasts (Wild type and *Bak*^*-/-*^*Bax*^*-/-*^) were cultured in DMEM supplemented with 10% FBS, 2 mM L-Glutamine, and standard antibiotics. WM1862, WM902B, WM3268, and WM793 lines were cultured in tumor specialized media with 2% FBS containing: 80% MCBD 153 medium (with trace elements & L-glutamine), 20% L-15 media (with L-glutamine), NaHCO_3_, and CaCl_2_. For flow cytometry based cell death studies, cells were seeded for 24 h, treated as described, floating and attached cells harvested, labeled with AnnexinV-FITC in binding buffer (10 mM HEPES pH 7.4, 150 mM NaCl, 5 mM KCl, 1 mM MgCl_2_, 1.8 mM CaCl_2_), and analyzed by flow cytometry as indicated. For clonogenic survival studies, cells were seeded for 24 h, treated as indicated for 24 h before changing the media, and cultured for seven days. Colonies were stained with 0.1% methylene blue and imaged. Colonies were then de-stained (20% methanol in 5% acetic acid), and the supernatant was measured for absorption at 568 nm for relative quantification of colony numbers.

### Western blot analyses

Whole-cell protein lysates were made from trypsinized cells, pelleted, resuspended in RIPA buffer supplemented with protease inhibitors (HALT, Pierce Biotechnology), incubated on ice for 10 min and centrifuged for 10 min at 21,000 × *g*. Lysates were then adjusted with RIPA buffer to equal the protein concentrations. Proteins (25–50 μg/lane) were subjected to SDS-PAGE before transferring to nitrocellulose by standard western conditions, blocked in 5% milk/TBST and primary antibodies (1:1000 in blocking buffer; incubated overnight at 4 °C). The secondary antibody (1:5000 in blocking buffer) was incubated at 25 °C for 1 h before standard enhanced chemiluminescence detection. For densitometric analyses, western blot films were scanned and processed using a CanoScan 5600 F scanner (Canon, USA) and MP Navigator EX software (Canon, USA). ImageJ software (Ref: Rasband, W.S., ImageJ, U. S. National Institutes of Health, Bethesda, Maryland, USA, https://imagej.nih.gov/ij/, 1997–2016) was used for densitometric measurement of the specific bands of interest. Values were normalized to β-Actin.

### Live-cell imaging

Cells were seeded on rat-tail collagen I coated plates for 24 h before indicated treatments. Mitochondria and nuclei were labeled with MitoTracker Green (100 nM) and Hoechst 33342 (20 μM) for 30 min at 37 °C, respectively. Phenol red free media supplemented with 10% FBS and 2 mM l-glutamine and antibiotics was used for all imaging performed on a Zeiss Imager.Z1 equipped with a N-Achroplan 40 × /0.75 water immersion lens and an AxioCAM MRm digital camera; images were captured using AxioVision 4.8 and Zeiss Zen software. At least 300 cells per condition were quantified. The Z-stack images were processed using Image J software (NIH) and the mitochondrial length was measured using NIS Elements software (Nikon, USA). Scale bars = 25 μm.

### Mitochondrial membrane potential by flow cytometry

Cells were seeded for 24 h, and treated as indicated. TMRE (100 nM) was added to the media, and the plates were incubated at 37 °C in the dark for 25 min. The cells were then trypsinized and analyzed by flow cytometry.

### Seahorse analysis

Cells were seeded in 200 μl DMEM complete media in XF96 plates (Seahorse Bioscience); plating densities: A375 4 × 10^3^, SK-MEL-28 8 × 10^3^, MeWo 5 × 10^3^, and treated as indicated. OCRs and ECARs were measured using the XF96 Extracellular Flux Analyzer and the XF Cell Mito Stress Test kit (Seahorse Bioscience). At the end of the assay, media was removed and cells were stained with methylene blue, de-stained, and the absorbance was measured at 668 nm using a plate reader (Synergy H1 Hybrid multi-mode micro-plate reader, BioTek). The OCR and ECAR measurements were normalized against the cell densities. Each experiment contained triplicate data points.

### Real-time live-cell analyses

Cells were seeded at 5,000 cells/well in 96 well tissue culture plates for 18–24 h before indicated treatments in phenol red-free growth media. For apoptosis assays, media contained recombinant Annexin V (1 μg/ml) labeled with either FITC or AlexaFluor 594 as described^47^; for DEVD assays, media contained CellEventTM Caspase-3/7 Green Detection Reagent (2 μM, Thermo Fisher Scientific); for mitochondrial membrane potential assays (Δ*φ*_M_), cells were loaded with TMRE (200 nM) for 30 min at 37 °C before indicated treatments. Cell images were captured at regular intervals, and fluorescent events were analyzed using the processing definitions listed below by the IncuCyte Zoom software. Fluorescent events were detected using the following IncuCyte Zoom filter cubes: Green Channel—Excitation 460 nm [440,480], Emission 524 nm [504,544]; Red Channel—Excitation 585 nm [565,605], Emission 635 nm [625,705]. Channel compensation was set at 5% Red from Green. Differences observed in the number of apoptosis events per cell line is due to differences in cell size, plating density, and proliferation rates that influence the maximal number of cells and subsequent apoptotic events per field.

### Real-time quantitative PCR

Total cellular RNA was extracted using an RNeasy kit (Qiagen) according to the manufacturer’s instructions. Total RNA (1 μg) was used to synthesize first-strand cDNA using the SuperScript^TM^ III First-Strand Synthesis System for RT-PCR (Invitrogen). Gene expression was analyzed using the SYBR Green detection system (FastStart Universal SYBR Green Master, Roche) and Applied Biosciences ViiA^TM^ 7 Real-Time PCR system, using the comparative *C*_T_ method. The expression of relevant genes was normalized to *β-actin* and *gapdh*. The following primer pairs (5′-3′) were used: *BAX*, TCAGGATGCGTCCACCAAGAAG and TGTGTCCACGGCGGCAATCATC; *BAK*, TTACCGCCATCAGCAGGAACAG and GGAACTCTGAGTCATAGCGTCG; *GAPDH*, GTCTCCTCTGACTTCAACAGCG and ACCACCCTGTTGCTGTAGCCAA; and *β-Actin*, CACCATTGGCAATGAGCGGTTC and AGGTCTTTGCGGATGTCCACGT.

### RNA interference

Plasmids for shRNA were purchased from Sigma-Aldrich (Mission^R^ shRNA; BAK and BAX). The pLKO empty vector and scrambled shRNA constructs were kindly provided by the laboratory of Dr. E. Premkumar Reddy (Icahn School of Medicine at Mount Sinai). The 293T cell line was used to produce retroviral and lentiviral particles for the generation of stable cell lines. Virus was harvested at 24 h and 48 h, pooled, and 0.45 μm filtered. Stable clones were generated using puromycin (0.4–0.8 μg/ml).

## Electronic supplementary material

Supplemental figures and legends
