# Supplementary material for: Dual suppression of inner and outer mitochondrial membrane functions augments apoptotic responses to oncogenic MAPK inhibition
Source: Cell Death Dis. 2018 Jan 18;9(2):29. doi: 10.1038/s41419-017-0044-1 (PMC5833689; doi:10.1038/s41419-017-0044-1)
Supplement: Supplementary file 1 — Supplemental figures and legends [file 41419_2017_44_MOESM1_ESM.docx]

**SUPPLEMENTAL FIGURES
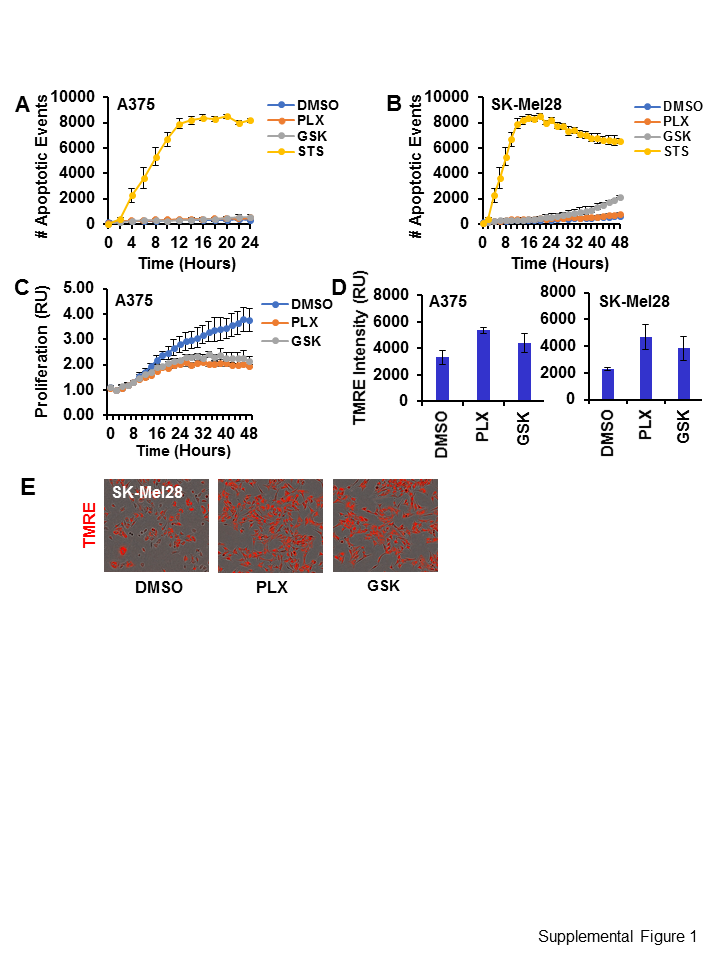
**

**Figure S1. Inhibition of oncogenic MAPK signaling deceases cell proliferation and promotes mitochondrial respiration but fails to induce apoptosis. (A-B)** A375 and SK-Mel28 were treated with PLX4032 (1 µM) or GSK1120212 (10 nM) for 24 h, and the number of apoptosis events was captured by IncuCyte ZOOM. Kinetic data for 24 h and 48h respectively are shown. Staurosporine (STS, 100 nM) is a positive control. **(C)** A375 was treated as in *A*, and confluency was quantified for 48 hours. Kinetic data is shown. **(D)** TMRE-loaded A375 and SK-Mel28 were treated as in *A* for 24 h, and Δφ*_M_* was captured by IncuCyte ZOOM. Endpoint data at 24 h are shown. **(E)** TMRE-loaded SK-Mel28 were treated as in *A* for 24 h, and Δφ*_M_* was visualized by IncuCyte Zoom. Images at 24 h are shown. All data are representative of at least triplicate experiments, and reported as ± S.D., as required.


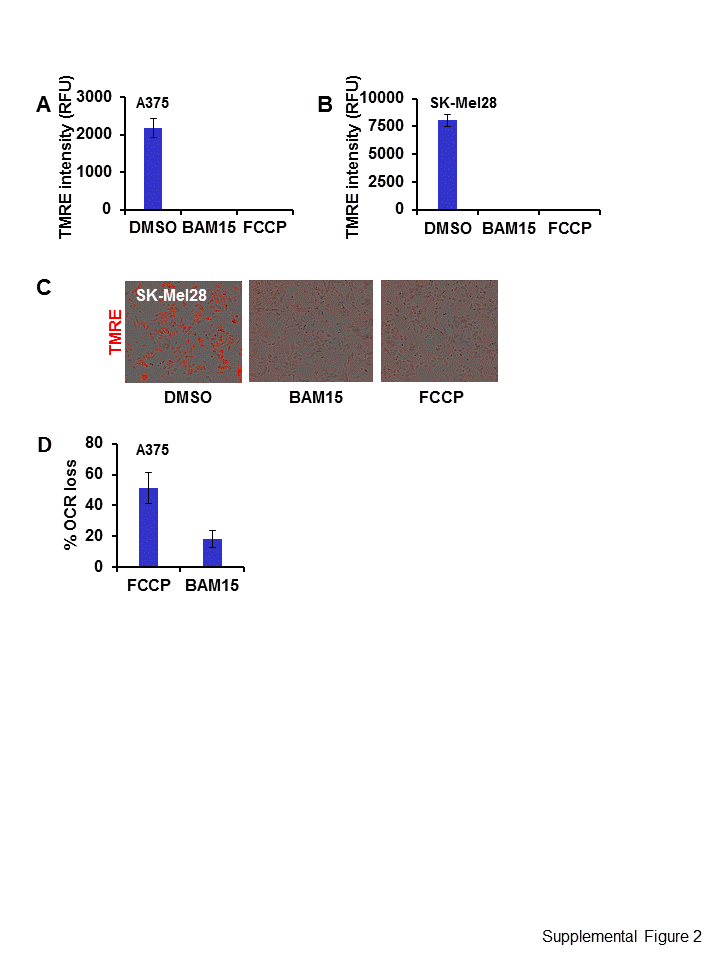


**Figure S2. BAM15 uncouples OXPHOS from electron transport in melanoma cell lines.** **(A-B)** A375 and SK-Mel28 cells were treated with FCCP (10 µM) or BAM15 (10 µM) for 2 h, loaded with TMRE (100 nM), and analyzed by flow cytometry. Quantification is shown. **(C)** SK-Mel28 were treated same as *A*, but analyzed by IncuCyte ZOOM and visualized. Images at 2 h are shown. **(D)** A375 were treated with FCCP (1 µM) or BAM15 (1 µM), and OCR was measured using a Seahorse flux analyzer. Percent loss of maximal OCR at 2 h is shown. All data are representative of at least triplicate experiments, and reported as ± S.D., as required.


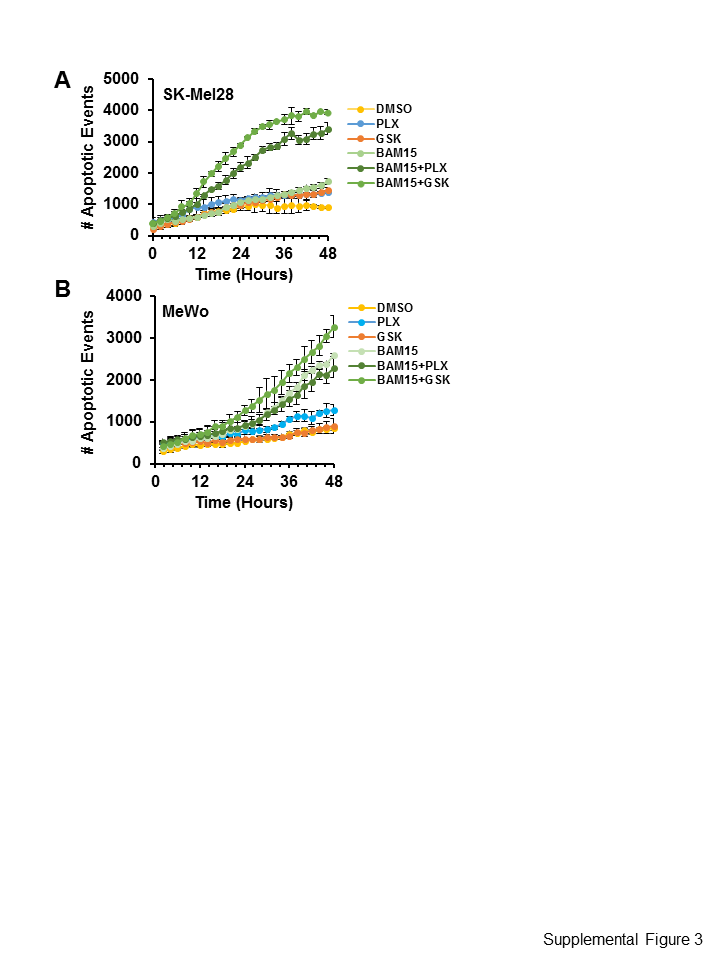


**Figure S3. BAM15 promotes cell death when oncogenic MAPK signaling is inhibited. (A-B)** SK-Mel28 and MeWo cells were treated with indicated doses of PLX4032 (0.5, 1, 10, 20 µM) or GSK1120212 (1, 5, 10, 25 nM) ± BAM15 (10 µM) and the kinetics of cell death were detected using an IncuCyte ZOOM. All data are representative of at least triplicate experiments, and reported as ± S.D., as required.

**
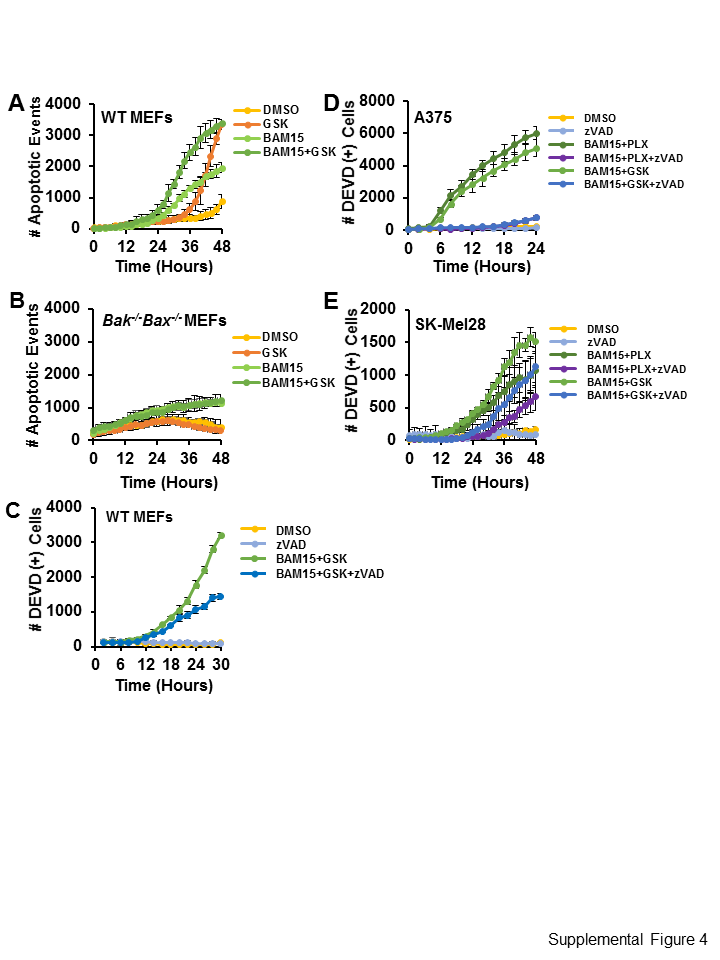
**

**Figure S4. Combined inhibition of OXPHOS and oncogenic MAPK signaling induces the mitochondrial pathway of apoptosis.** **(A-B)** Wt and *Bak^-/-^Bax^-/-^* MEFs were treated with GSK1120212 (10 nM) ± BAM15 (10 µM) for 48 h, and apoptosis was detected by AnnexinV-FITC staining with an IncuCyte ZOOM. Kinetic data are shown. **(C)** Wt MEFs were treated with GSK1120212 (10 nM), BAM15 (10 µM), and ± zVADfmk (100 µM) for 30 h, and caspase activity was detected by DEVD-FITC with an IncuCyte Zoom. Kinetic data over 30 h are shown. **(D-E)** A375 and SK-Mel28 were treated with indicated combinations of PLX4032 (1 µM) or GSK1120212 (10 nM), BAM15 (10 µM), ± zVAD-fmk (100 µM), and caspase activity was detected by DEVD-FITC with an IncuCyte ZOOM. Kinetic data for 24 and 48h respectively are shown. All data are representative of at least triplicate experiments, and reported as ± S.D., as required.


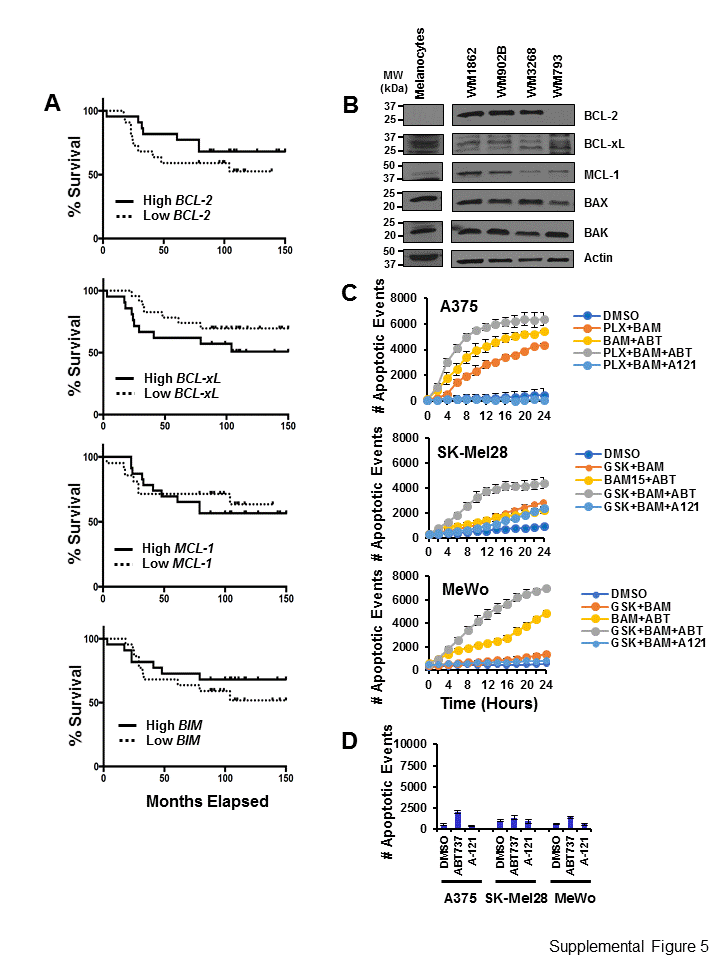


**Figure S5. Dual suppression of inner and outer mitochondrial membrane functions augments pro-apoptotic responses to oncogenic MAPK signaling inhibition.** **(A)** Kaplan-Meyer curves representing the 5-year overall survival for *BCL-2* (P = 0.0417), *BCL-xL* (P = 0.0004), *MCL-1* and *BIM* across primary melanoma samples (n = 51). ‘High’ (n = 24) and ‘low’ samples (n = 27) were defined based on high (positive) and low (negative) z-scores generated from gene expression data. Log-rank test was utilized to determine statistical significance. **(B)** Primary melanocytes, WM1862, WM963B, WM3268, and WM793 whole cell lysates were subjected to SDS-PAGE and western blot for indicated proteins. Actin is the loading control. **(C)** A375, SK-Mel28, and MeWo were treated with the indicated combinations of drugs: PLX4032 (1 µM), GSK1120212 (10 nM), BAM15 (10 µM), ABT737 (1 µM), and A1210477 (1 µM); apoptosis was detected by AnnexinV-FITC staining with an IncuCyte ZOOM. Kinetic data for 24 h are shown. **(D)** A375, SK-Mel28, and MeWo were treated with ABT737 (1µM) or A121 (1µM); apoptosis was detected by AnnexinV-FITC staining with an IncuCyte ZOOM. Endpoint data at 36 h are shown.
